# Intermittent hypoxia therapy ameliorates beta-amyloid pathology via TFEB-mediated autophagy in murine Alzheimer's disease

**DOI:** 10.1186/s12974-023-02931-6

**Published:** 2023-10-20

**Authors:** Xueting Wang, Yuqi Xie, Guijuan Chen, Yapeng Lu, Dan Wang, Li Zhu

**Affiliations:** Institute of Special Environmental Medicine, Co-Innovation Center of Neuroregeneration, Nantong University, No. 9, Seyuan Road, Chongchuan District, Nantong, 226009 Jiangsu China

**Keywords:** Alzheimer's disease, Transcription factor EB, Plaque-associated microglia, Beta-amyloid degradation, Autophagy

## Abstract

**Background:**

Alzheimer's disease (AD) is the most prevalent neurodegenerative disorder. Impaired autophagy in plaque-associated microglia (PAM) has been reported to accelerate amyloid plaque deposition and cognitive impairment in AD pathogenesis. Recent evidence suggests that the transcription factor EB (TFEB)-mediated activation of the autophagy–lysosomal pathway is a promising treatment approach for AD. Moreover, the complementary therapy of intermittent hypoxia therapy (IHT) has been shown to upregulate autophagy and impart beneficial effects in patients with AD. However, the effect of IHT on PAM remains unknown.

**Methods:**

8-Month-old APP/PS1 mice were treated with IHT for 28 days. Spatial learning memory capacity and anxiety in mice were investigated. AD pathology was determined by the quantity of nerve fibers and synapses density, numbers of microglia and neurons, Aβ plaque deposition, pro-inflammatory factors, and the content of Aβ in the brain. TFEB-mediated autophagy was determined by western blot and qRT-PCR. Primary microglia were treated with oligomeric Aβ 1–42 (oAβ) combined with IHT for mechanism exploration. Differential genes were screened by RNA-seq. Autophagic degradation process of intracellular oAβ was traced by immunofluorescence.

**Results:**

In this study, we found that IHT ameliorated cognitive function by attenuating neuronal loss and axonal injury in an AD animal model (APP/PS1 mice) with beta-amyloid (Aβ) pathology. In addition, IHT-mediated neuronal protection was associated with reduced Aβ accumulation and plaque formation. Using an in vitro PAM model, we further confirmed that IHT upregulated autophagy-related proteins, thereby promoting the Aβ autophagic degradation by PAM. Mechanistically, IHT facilitated the nuclear localization of TFEB in PAM, with TFEB activity showing a positive correlation with Aβ degradation by PAM in vivo and in vitro. In addition, IHT-induced TFEB activation was associated with the inhibition of the AKT–MAPK–mTOR pathway.

**Conclusions:**

These results suggest that IHT alleviates neuronal damage and neuroinflammation via the upregulation of TFEB-dependent Aβ clearance by PAM, leading to improved learning and memory in AD mice. Therefore, IHT may be a promising non-pharmacologic therapy in complementary medicine against AD.

**Graphical Abstract:**

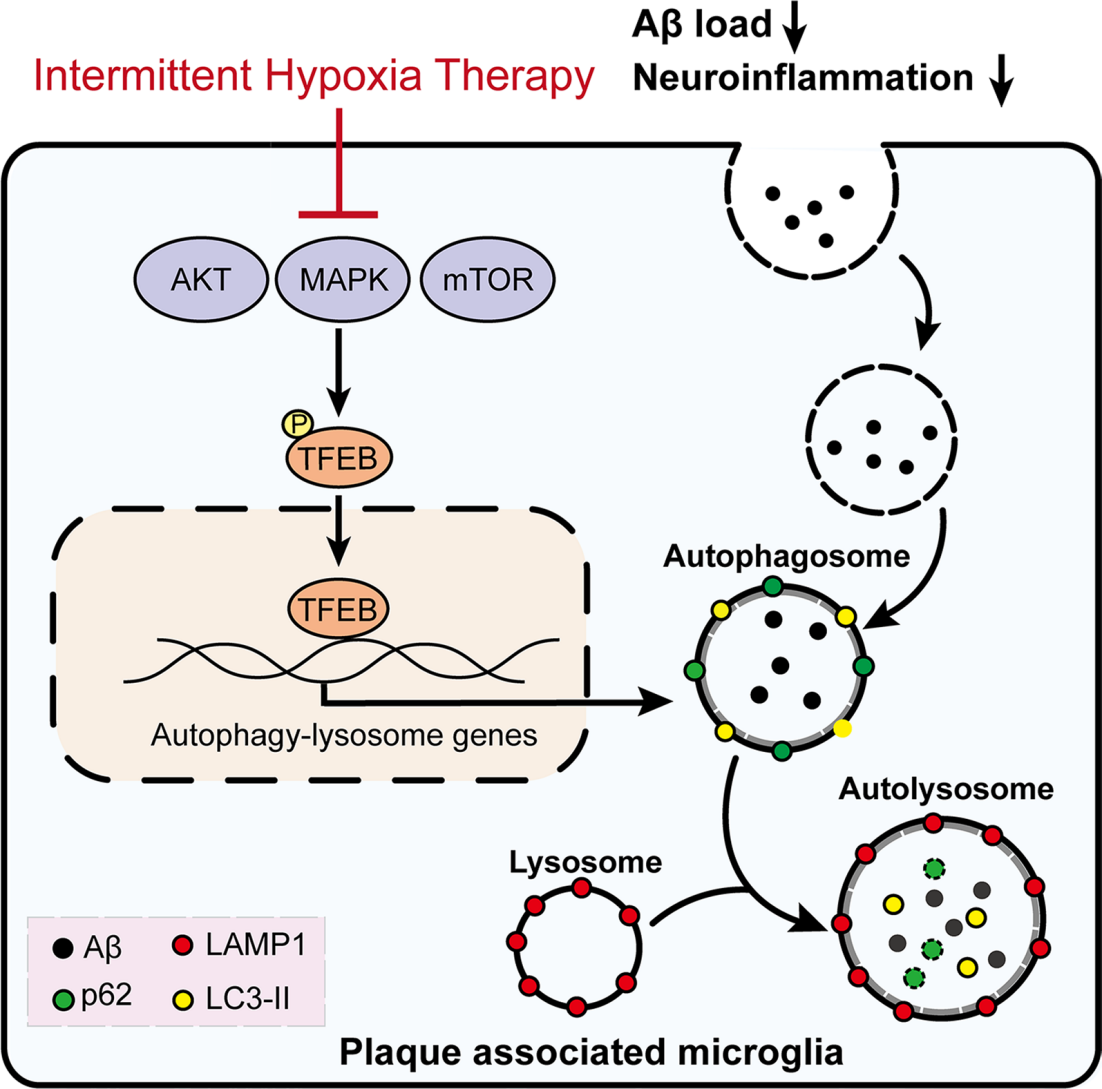

**Supplementary Information:**

The online version contains supplementary material available at 10.1186/s12974-023-02931-6.

## Introduction

Alzheimer's disease (AD), which is the most common neurodegenerative disease, is characterized by two major pathological brain lesions, i.e., amyloid plaques (composed mainly of beta-amyloid [Aβ]) and neurofibrillary tangles (primarily comprising hyperphosphorylated tau). The accumulation and aggregation of Aβ and tau are detrimental to neurons, eventually leading to neurodegeneration. Therefore, stimulating the clearance of these neuropathological aggregates is an attractive therapeutic strategy for AD [1, 2]. Moreover, Aβ is suggested to drive tau pathology, with the lowering of Aβ to certain levels showing beneficial effects in clinical treatment [3]. However, no effective medication currently exists for AD. Nevertheless, drugs such as aducanumab Aduhelm (approved by the United States Food and Drug Administration in 2021) have been demonstrated to reduce Aβ plaques [4]; however, their effectiveness and safety remain unclear [5].

Accumulating evidence suggests that the phagocytic and degradative activities of microglia are crucial in eliminating Aβ from the brain [6–8]. Amyloid precursor protein is processed into Aβ monomers by β/γ-secretases in neurons. Aβ monomers are the most abundant form in the brain and forms Aβ oligomers (oAβs) and fibrillar Aβ, which further lead to the formation of amyloid plaques. Among these three types of Aβ, oAβ is considered the most neurotoxic form, causing glial cell activation and neuroinflammation [9]. In early stage AD, oAβs enhance Aβ autophagic degradation by upregulating LC3-associated endocytosis (LANDO) in microglia [10]. These oAβs are then internalized by microglia via the receptor TREM2 and degraded in the autolysosomes [11]. Thus, LANDO is a key process in maintaining Aβ homeostasis in the brain.

In late-stage AD, plaque-associated microglia (PAM) show markedly impaired autophagic function, resulting in exacerbated plaque deposition and cognitive damage [12–14]. In particular, PAM primarily uses the autophagy–lysosomal pathway (ALP) for Aβ degradation, wherein the transcription factor EB (TFEB) is the main regulator [15, 16]. TFEB is a transcriptional autophagy regulator activated by dephosphorylation and nuclear translocation, a process inhibited by signaling kinases, such as mTOR, MAPK, and AKT [17]. TFEB activation by oxidative stress induces autophagy upregulation, which in turn confers neuroprotection, thus making it a potential therapeutic target. [18]. Moreover, increasing research suggests that TFEB activation enhances microglia-mediated clearance of Aβ plaques, thereby improving cognitive function via autophagy upregulation in the brains of AD animals [19–21]. Therefore, targeting TFEB activation appears to be an effective AD therapy.

Intermittent hypoxia therapy (IHT) improves an organism's physiological and biochemical adaptations to hypoxia by inducing intermittent moderate hypoxia in the organism. Clinical studies have found that IHT boosts exercise tolerance in patients with chronic cardiovascular disease, chronic obstructive pulmonary disease, or metabolic disease [22]. These IHT benefits may be associated with optimizing mitochondrial metabolism and preventing reactive oxygen species (ROS) production [23]. Specifically, IHT upregulates autophagy through the mTOR pathway, leading to enhanced neuroplasticity, reduced myocardial ischemic injury, improved vascular endothelial function, and decreased endoplasmic reticulum stress and apoptosis [24, 25]. In line with these findings, Schega et al. observed that IHT positively affected cognitive function in a study of an older population [26].

Studies have reported that IHT ameliorates certain diseases by activating the autophagy pathway [24, 25]. Song et al. showed that IHT resisted apoptosis in pancreatic β-cells by inducing autophagy and activating the endoplasmic reticulum stress-related PERK/eIF2α/ATF4 pathway [27]. Chi et al. showed that IHT administered to rats at 8 h per day for a total of 35 days ameliorates myocardial injury by upregulating autophagy [28]. IHT is known to induce the protective oxidative response of cells, with mild oxidative stress being shown to be neuroprotective via the induction of TFEB-mediated autophagy [18, 25, 29]. TFEB, a main transcription factor regulating the ALP [15, 19, 30], enhances Aβ clearance by PAM [19, 31]. Thus, we hypothesize that IHT has the potential to improve autophagy by upregulating lysosomal function.

Recent studies have revealed that IHT provides superior protection against AD by reducing brain Aβ deposition and alleviating cognitive function [32, 33], indicating that IHT is a promising strategy for symptomatic AD treatment. Therefore, elucidating the underlying mechanism of IHT in ameliorating AD to promote the clinical use of IHT in AD treatment is crucial. Here, we found that IHT improved PAM function by activating TFEB, resulting in enhanced Aβ autophagic clearance and consequently reduced neural damage and improved cognitive function in AD models.

## Materials and methods

### Animals and treatments

Specific pathogen-free (SPF), male APP/PS1 (B6C3-Tg [APPswe, PSEN1de9] 85Dbo/J [005864]) and wild-type littermate control C57BL/6 J mice on a C57BL6/J background aged 6–8 months were purchased form Nanjing Junke Bioengineering Corporation, Ltd. (Nanjing, China; certification number SCXK 2020-0009). Animals were maintained at 23 ± 2 °C under 45–60% humidity and a standard 12/12-h light/dark cycle. For TFEB activation, 6-month-old mice were orally administered TFEB activator 1 (TA1, 10 mg/kg/day; MedChemExpress, HY-135825, CAS: 39777-61-2) or vehicle (corn oil) for 3 months [31]. During IHT, mice were placed in a 60 cm × 30 cm × 25 cm chamber with rapidly adjustable oxygen levels. In this therapy, the oxygen concentration in the chamber was initially reduced to 8% within 30 s and maintained for 8 min by introducing compressed nitrogen, followed by compressed oxygen to restore the oxygen concentration to 21% within 30 s and sustained for 8 min. A total of 10 cycles of this hypoxia–normoxia exposure was performed daily for 28 days between 09:00 and 11:00. The IHT protocol was modified from a procedure that has proven to be neuroprotective in 5 × FAD mice [33]. After IHT, the mice were euthanized and perfused with 0.9% saline via the left ventricle to remove the blood.

### Morris water maze (MWM) test

MWM tests for spatial learning-memory behavior were conducted as previously described by Zha et al. [34]. The maze was divided into four equal quadrants: the northeast, southeast, southwest, and northwest. Visual cues were posted on the laboratory walls around the maze to facilitate the spatial learning of the platform's location. After IHT, mice were placed in a 150-cm circular pool with water temperature maintained at 21 ± 1 °C. A circular escape platform (10 cm in diameter) was placed in the middle of the southwest quadrant at 1.5 cm below the water surface. The water was made opaque with a non-toxic, white pigment. The mice were allotted 60 s to locate the platform and were allowed to stay on it for 20 s. The mice were trained four times per day for 5 days. On the 6th day, the hidden platform was removed from the destination quadrant. The mice were then released in the northeast quadrant and allowed to swim freely for 60 s. Video recordings were used to analyze and record the swimming trajectory of each mouse, along with measurement of the time required by the mice to initially find the escape platform, time spent in the target quadrant, and the average swimming speed.

### Open field test (OFT)

The OFT was used to assess the anxious behavior of the mice and was performed according to a prior study by Kraeuter et al. [35]. After IHT treatment, the mice were individually placed in a white polyvinyl chloride box (40 cm × 40 cm × 40 cm) and allowed to move freely for 5 min. An overhead camera was used to capture the movement of the test animal in the peripheral (15 cm from the wall) and central zones (25 cm × 25 cm). The number of entries and time spent in the central area by the mice were also recorded.

### Elevated plus maze (EPM) test

The EPM test was employed to measure anxiety-related behavior in the mice and was conducted as previously described [36]. Briefly, the mice were positioned at the junction of the four arms (two open and two closed arms) of a maze (35 cm × 5 cm, 50 cm from the ground) facing an open arm and allowed to freely explore the maze for 5 min. Their behavior was recorded using a video camera above the maze. In addition, the entries and time spent by the mice in the open and closed arms were recorded.

### Nissl staining

Brain sections (30 μm) of the euthanized mice were fixed using 4% neutral paraformaldehyde and stained with 1% tar violet. The samples were subsequently dehydrated using an ethanol gradient and transparent xylene. The samples were imaged using a Leica DM4000B microscope.

### Primary microglia culture and IHT for the microglia

*Primary microglia culture* Based on previously described protocols [37–39], primary microglial cells were obtained from the cerebral cortices of 3-day-old C57BL/6 mice. After removing the meninges, cortical tissue was digested using 0.05% trypsin. The separated cells were then cultured in Dulbecco's modified Eagle's medium-F12 supplemented with 10% fetal bovine serum, 5 ng/ml of Granulocyte–macrophage colony-stimulating factor (STEMCELL Technologies, 78017), and penicillin/streptomycin (100 U/ml and 100 mg/ml, respectively) at 37 °C in a 5% CO_2_ humidified incubator. After 10 days, the mixed cell population was dominated by astrocytes and formed a fused trophoblast. The microglia gradually proliferated and floated in the supernatant. Finally, the cells from the supernatant were harvested and seeded in 35-mm confocal dishes.

*IHT for the primary microglial cells* The cell cultures were placed in the same intermittent hypoxia chamber used for the experimental mice. The cells were exposed to 21% oxygen (8 min) and 8% oxygen (8 min) for 10 cycles*.*

### Construction of the in vitro PAM model

Lyophilized Aβ1–42 (Anaspec, AS-20276) were dissolved in PBS and incubated overnight at 4 °C to form oligomers (oAβ) [40]. The microglial cells were treated with 1 μM of oAβ for 0, 3, 6, 9, 12, 18, and 24 h. The residual oAβ in the supernatant was detected via ELISA to evaluate the ability of the microglia to metabolize oAβ. Phagocytosis ability and autophagy flux were determined as described below.

### TA1 treatment

TA1 (CAS 39777-61-2) purchased from MedChemExpress (HY-135825, 99.69% purity) was dissolved in DMSO to yield a final concentration of 25 mg/ml. It was further diluted tenfold to 2.5 mg/ml with corn oil for animal treatment. In the case of the mice treatment, 6-month-old APP/PS1 mice were administered TA1 orally at 10 mg/kg for 3 months. For cell treatment, TA1 was further diluted to 1 mM in DMSO and directly added to the culture medium to produce a working concentration of 1 μM.

### Bafilomycin A1 treatment

Bafilomycin A1 (CAS 88899-55-2) was procured from MedChemExpress (HY-100558, 98.77% purity). The colocalization of Aβ and LAMP1 in PAM in vitro was examined by adding 100 nM of bafilomycin A1 to the culture medium at 1 h before cell fixation.

### Detection of the activation of TFEB pathway

TFEB pathway activation were determined by transcriptome screening and qRT-PCR validation. After IHT treatment, total RNA was isolated with TRIzol reagent. The purified total RNA was then submitted to Gene Denovo Biotechnology Co. for RNA-seq. Furthermore, purified total RNA was reverse-transcribed using the HiScript III 1st Strand cDNA Synthesis Kit (Vazyme Biotech, R312-02), according to the manufacturer's instructions and qRT-PCR was performed via the AceQ qPCR SYBR Green Master Mix (Vazyme Biotech, Q141-02).

To further confirm the relationship between IHT and TFEB, primary microglia were transfected by lentivirus expressing mouse sh*Tfeb* (#1, #2 and #3) to silence TFEB expression. Aβ-555 endocytosis and degradation, and autophagy level were determined.

### Lentivirus transfection

After 70% fusion of the trophoblast cells (approximately 7 days of primary culture), the cells were incubated with 8 × 10^7^ TU of lentiviruses per 25 cm^2^ culture flask and allowed to grow until microglia production. The lentivirus transfection efficiency was approximately 70% in the harvested microglia. Lentivirus expressing LC3-GFP was used to observe autophagosomes. In addition, lentivirus expressing mouse sh*Tfeb* (#1 target sequence: CGGCAGTACTATGACTATGAT, 2# target sequence: GCGGCAGAAGAAAGACAATCA, and #3 target sequence: GGAGATGACTAACAAGCAGCT) was used to silence TFEB expression.

### Aβ-555 endocytosis and degradation assay

*Confocal imaging* Primary microglial cells were incubated with 1 μg/ml of oligomer Aβ1–42, HiLyte™ Fluor 555 (Aβ-555) (Anaspec, AS-60480-01) for 30 min, followed by fixation with 4% paraformaldehyde for endocytosis analysis. In the degradation assay, the microglia were further chased with fresh medium and incubated for 30 min, followed by immediate fixation with 4% paraformaldehyde. Finally, the microglial cells were counterstained with DAPI, and confocal imaging was performed (552 nm; Leica SP8). Positive signals within a single cell were estimated using FIJI Image J (National Institutes of Health).

*Flow cytometry* The harvested microglial cells were incubated directly with Aβ-555 for 30 min. The ratio of positive cells containing the HiLyte 555 signal was determined using flow cytometry.

### Immunofluorescence staining

Tissue sections (40 μm) and cultured cells were fixed with 4% paraformaldehyde. The samples were subsequently permeabilized with 0.5% Triton X-100. The samples were then blocked in 10% donkey serum, followed by incubation with the following antibodies: anti-MAP2 (Sigma-Aldrich, M4403), anti-Aβ1–16 (BioLegend, SIG-39300), anti-Iba1 (Abcam, ab5076), anti-TFEB (Proteintech, 13372-1-AP), anti-LC3A/B (Cell Signaling Technology, 12741S), anti-LAMP1 (Abcam, ab24170), and anti-synaptophysin (Cell Signaling Technology, 9020). The binding of the primary antibodies was visualized using Alexa Fluor 555-conjugated donkey anti-rabbit IgG (Thermo Fisher Scientific, A31572), Alexa Fluor 488-conjugated donkey anti-mouse IgG (Thermo Fisher Scientific, A21202), or Alexa Fluor 647-conjugated donkey anti-goat IgG (Abcam, ab150131). Finally, the samples were counterstained with DAPI, and confocal microscopy (Leica Thunder or Leica SP8 confocal microscope) was used to capture the fluorescence images.

### RNA isolation and qRT-PCR

Total RNA from the samples was isolated with a TRIzol reagent. Purified total RNA was reverse-transcribed using the HiScript III 1st Strand cDNA Synthesis Kit (Vazyme Biotech, R312-02), according to the manufacturer's instructions. The purified total RNA was then submitted to Gene Denovo Biotechnology Co. for RNA-seq. Furthermore, qRT-PCR was performed via the AceQ qPCR SYBR Green Master Mix (Vazyme Biotech, Q141-02).

All primers used for qRT-PCR were designed as follows.

*Lamp1*, forward: 5′-CAGCACTCTTTGAGGTGAAAAAC-3′, reverse: 5′-ACGATCTGAGAACCATTCGCA-3′.

*Il1b*, forward: 5′-TGCCACCTTTTGACAGTGATG-3′, reverse: 5′-TGATGTGCTGCTGCGAGATT-3′.

*Tnf*, forward: 5′-AAGCCTGTAGCCCACGTCGTA-3′, reverse: 5′-GGCACCACTAGTTGGTTGTCTTTG-3′.

*Tgfb*, forward: 5′-TGATACGCCTGAGTGGCTGTCT-3′, reverse: 5′-CACAAGAGCAGTGAGCGCTGAA-3′.

*Wipi2*, forward: 5′-TGCTGGTAGGAGCATCAGATGG-3′, reverse: 5′-TCACTGGTCGTCTCCATACTGC-3′.

*Vps11*, forward: 5′-ATCGGCAGTCTCTGGCTAATGC-3′, reverse: 5′-GGACCTTGATGGCTGTCTCTAC-3′.

*Vps18*, forward: 5′-AAGTGAGCCCAACCGTGTGGAA-3′, reverse: 5′-AAAGGACCTCGGTGCTACTCAG-3′.

*Actb*, forward: 5′-CATCCGTAAAGACCTCTATGCCAAC-3′, reverse: 5′-ATGGAGCCACCGATCCACA-3′.

The relative amount of gene expression was calculated using ΔCt values, where Ct represents the threshold cycle of PCR.

### Estimation of Aβ1–42 and Aβ1–40 levels

Soluble and insoluble proteins from the mice brains, supernatant, or cultured microglia were diluted to 21% using PBS to determine Aβ1–42 and Aβ1–40 levels via specific ELISA methods. The test procedures were performed according to the manufacturer's instructions for the Human Aβ1–40 ELISA Kit (Immunoway Biotechnology, KE1389) and Human Aβ1–42 ELISA Kit (Immunoway Biotechnology, KE1390). The optical density was measured at 450 nm. In addition, the standard curve was created using a four-parameter logistic curve fit with Origin 8.0 software (https://www.originlab.com).

### Protein isolation and western blot

The samples were lysed in RIPA buffer to obtain soluble proteins. The precipitates were further cleaved with RIPA buffer containing SDS and urea to obtain insoluble proteins. The protein concentration was determined via bicinchoninic acid assay. The proteins were then isolated and transferred to polyvinylidene fluoride membranes. Next, the membranes were incubated with primary antibodies, including anti-Aβ1–16 (BioLegend, SIG-39300), anti-TFEB (Proteintech, 13372-1-AP), anti-phospho-MAPK (Cell Signaling Technology, 4370), MAPK (Cell Signaling Technology, 4695), anti-p-Akt (Cell Signaling Technology, 4060 T), anti-Akt (Cell Signaling Technology, 4685 s), anti-ribosomal protein S6 (RPS6; Santa Cruz Biotechnology, sc-74459), anti-p-RPS6 (Cell Signaling Technology, 2211), anti-mTOR (Santa Cruz Biotechnology, sc-517464), anti-p-mTOR (Santa Cruz Biotechnology, sc-293133), anti-p62 (Proteintech, 18420-1-AP), anti-LC3A/B (Cell Signaling Technology, 12741S), anti-LAMP1 ([Santa Cruz Biotechnology, sc-20011] or [Abcam, ab24170]), and anti-β-actin (Sigma-Aldrich, A5316). The binding of the primary antibodies was visualized using HRP-conjugated secondary antibodies. Furthermore, grayscale analysis was conducted using FIJI ImageJ (National Institutes of Health).

### Statistical analysis

GraphPad Prism version 8 (GraphPad) was used to analyze the data. A Student's *t* test and one- or two-way ANOVA followed by Dunnett's multiple comparisons were applied for statistical assessment. Data are presented as mean ± Standard Error of Measurement (SEM). The significance levels for all the graphs were as follows: *P < 0.05, **P < 0.01, and ***P < 0.001. *P* values with no statistical difference have been marked in the graph.

## Results

### IHT ameliorates cognitive function in APP/PS1 mice

**Fig. 1:**
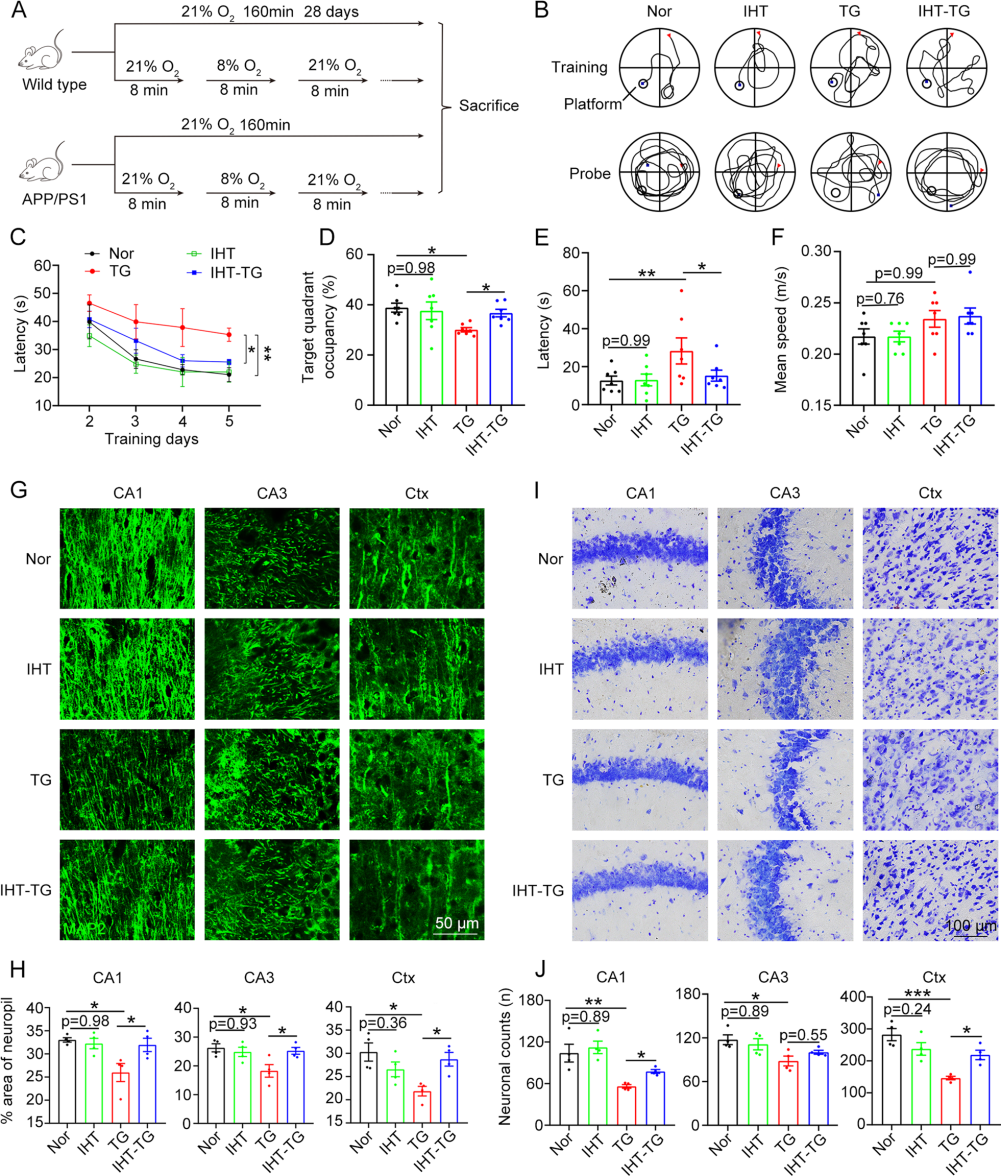
IHT improves learning and memory and ameliorates neural damage in APP/PS1 mice. **A** Design of the IHT system is illustrated. In the IHT procedure, 8-month-old APP/PS1 and wild-type littermate control mice were treated with 10 alternating cycles of 21% O_2_ (8 min) and 8% O_2_ (8 min) daily (160 min/day) for 28 days. **B** After IHT, the mice were trained in the MWM for 5 consecutive days. The mice swimming trajectories in the water maze on the 5th day (training test) and on the 6th day after the escape platform was removed (probe test) were recorded. **C** Latencies to reach the escape platform from the contralateral quadrant were recorded during the continuous 5-day training. **D** In the probe test, the dwelling time in the target quadrant was calculated as the percentage of the total time. **E** During the probe test, the latency to reach the target quadrant for the first time was recorded. **F** In the probe test, the average swimming speed was obtained by calculating the total swimming distance divided by the total duration. **G** Axonal labeling in the brain sections of the IHT-administered mice was performed by staining with anti-MAP2 antibodies. Scale bar = 50 μm. **H** Axonal integrity was determined by calculating the relative area of MAP2 signals in the hippocampal CA1 and CA3 regions and the cortex. **I** Neurons in the brain sections were labeled with Nissl staining. Scale bar = 100 μm. **J** Number of neurons in the hippocampal CA1 and CA3 region and the cortex was estimated by counting the blue particles. n = 7 in panels C–F; n = 4 in panels H–J. *P < 0.05, **P < 0.01, and ***P < 0.001 by two-way ANOVA. *Ctx* cortex, *Hipp* hippocampus, *TG* APP/PS1 transgenic mice

We administered IHT (8% O_2_ for 8 min and 21% O_2_ for 8 min, 10 cycles/day) in 8-month-old APP/PS1 and wild-type (WT) littermate control mice for 28 days (Fig. 1A). The Morris water maze (MWM) results revealed that the swimming trajectories to reach the platform in the training trials were longer in the APP/PS1 mice than in the WT mice, while IHT-treated APP/PS1 mice demonstrated a shorter swimming route to the target quadrant (Fig. 1B). In the probe trials, the WT mice remained in the target quadrant, whereas the APP/PS1 mice exhibited indiscriminate movement to all quadrants. In contrast, the IHT-treated APP/PS1 mice displayed extended dwelling in the target quadrant (Fig. 1D). Furthermore, the latency to reach the escape platform for the first time was significantly longer in the APP/PS1 mice than in the WT mice, whereas IHT significantly shortened the latency time in the APP/PS1 mice during the 5-day training duration (Fig. 1C). Consistent with these findings, the results of the probe trials on the 6^th^ day showed that the APP/PS1 mice had a shorter dwelling time in the target quadrant and a longer latency time than the WT mice, and these values were largely reversed by IHT (Fig. 1D, E). However, the swimming speed was similar across all mice groups, suggesting that IHT has little effect on the muscle (Fig. 1F). Moreover, the open field and elevated plus maze tests indicated that IHT did not improve the anxiety of the mice (Additional file 1: Fig. S1). All these results suggest that the learning and memory of 8-month-old APP/PS1 mice were significantly impaired and that IHT effectively improved the cognitive function of these mice.

We further investigated whether the behavioral improvement of the IHT-treated APP/PS1 mice was associated with changes in their neural structures. Our examination showed a significant loss of axons and dendrites in the brain of the APP/PS1 mice. Furthermore, IHT significantly blocked this neuronal loss in the hippocampal CA1 and CA3 and cortical regions of the APP/PS1 mice (Fig. 1G, H, Additional file 1: Fig. S2). In addition, compared with the WT mice, the APP/PS1 mice exhibited a significantly reduced number of brain neurons, which was reversed after IHT (Fig. 1I, J). Considering these findings, IHT may improve cognitive function by mitigating neuronal impairment in APP/PS1 mice.

### IHT alleviates Aβ pathology and neuroinflammation in APP/PS1 mice

**Fig. 2:**
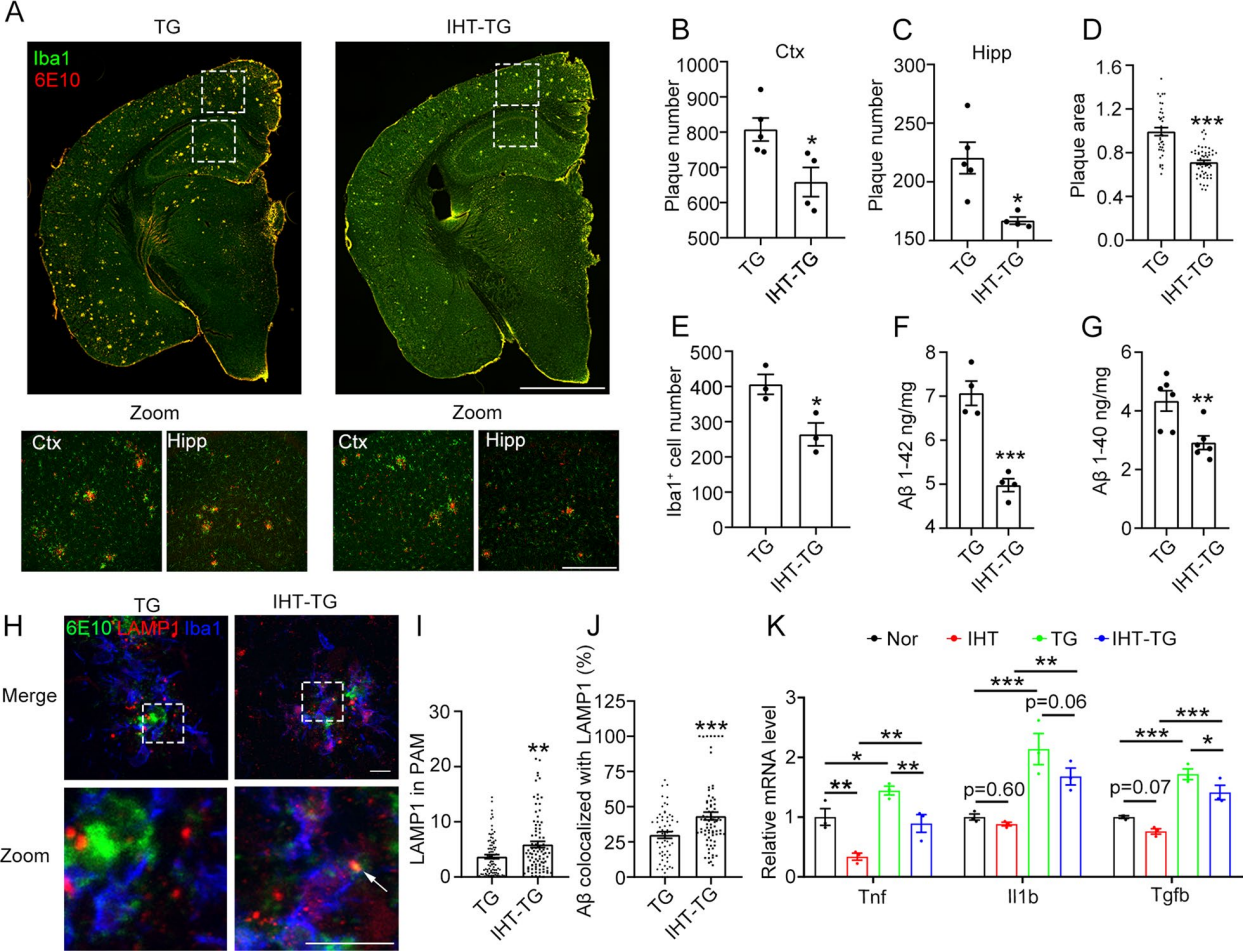
IHT reduces plaque accumulation, enhances Aβ degradation, and attenuates the expression of pro-inflammatory factors. During IHT, 8-month-old APP/PS1 mice underwent IHT for 28 days. **A** Mice brain sections were probed with anti-Aβ1–16 (6E10) and anti-Iba1 antibodies to detect Aβ plaques and microglia. The cortex and CA1 region of the hippocampus are circled and zoomed in. Scale bar = 2 mm or 300 μm. **B,** **C** Total of 12 evenly spaced brain slices were acquired from each mouse, and the number of plaques in the hippocampus and cortex was quantified by counting the 6E10 signals. n = 5. **D** Plaque area was obtained by measuring the average area of the 6E10 signals. n > 40. **E** Microglia number was estimated by counting all Iba1^+^ cells in the hemispheres. n = 3. **F,** **G** Aβ1–42 and Aβ1–40 concentrations in the brain tissues were determined via ELISA. n = 4 and 6. **H** Brain sections were co-stained with anti-LAMP1, anti-Iba1, and anti-Aβ1–16 (6E10) antibodies to observe the degraded Aβ in PAM. Scale bar = 10 μm. **I** LAMP1 in PAM were calculated by measuring the mean intensity of LAMP1 in Iba1^+^6E10^+^ cells. n > 90. **J** Colocalization ratio of Aβ with LAMP1 in Iba1^+^ cells were determined using Manders' colocalization coefficients. n > 50. **K** mRNA levels of *Il1b*, *Tnfa*, and *Tgfb* in the hippocampus were obtained via qRT-PCR. n = 4. *P < 0.05, **P < 0.01, and ***P < 0.001 using Student's *t* test. *Ctx* cortex, *Hipp* hippocampus, *TG* APP/PS1 transgenic mice

To further confirm whether IHT-induced cognitive function improvement in the APP/PS1 mice was related to changes in Aβ pathology, we co-labeled Aβ plaques with the microglial marker Iba1 in mouse brain sections (Fig. 2A). Our findings revealed that IHT significantly reduced the number and area of Aβ plaques in the cortex and hippocampus (Fig. 2B–D), which was accompanied by fewer activated microglia (Fig. 2E). Furthermore, IHT significantly reduced the levels of Aβ1–40 and Aβ1–42 (Fig. 2F, G), the most common Aβ subtypes, suggesting that IHT effectively suppresses Aβ plaque deposition.

We additionally explored the effect of IHT on Aβ clearance by PAM. As demonstrated in Fig. 2H, I, IHT significantly upregulated lysosome formation in PAM, along with significantly increasing the colocalization of the lysosomes with Aβ (Fig. 2J), suggesting that IHT promotes the lysosomal clearance of Aβ in PAM. Furthermore, IHT alleviated neuroinflammation in the APP/PS1 mice, as evidenced by the reduced levels of pro-inflammatory factors IL-1β and TNF-α and upregulated anti-inflammatory factor TGF-β (Fig. 2K). Therefore, IHT enhances Aβ clearance by PAM and reduces Aβ plaque accumulation and neuroinflammation.

### IHT-stimulated Aβ clearance is accompanied by autophagy activation in PAM

**Fig. 3:**
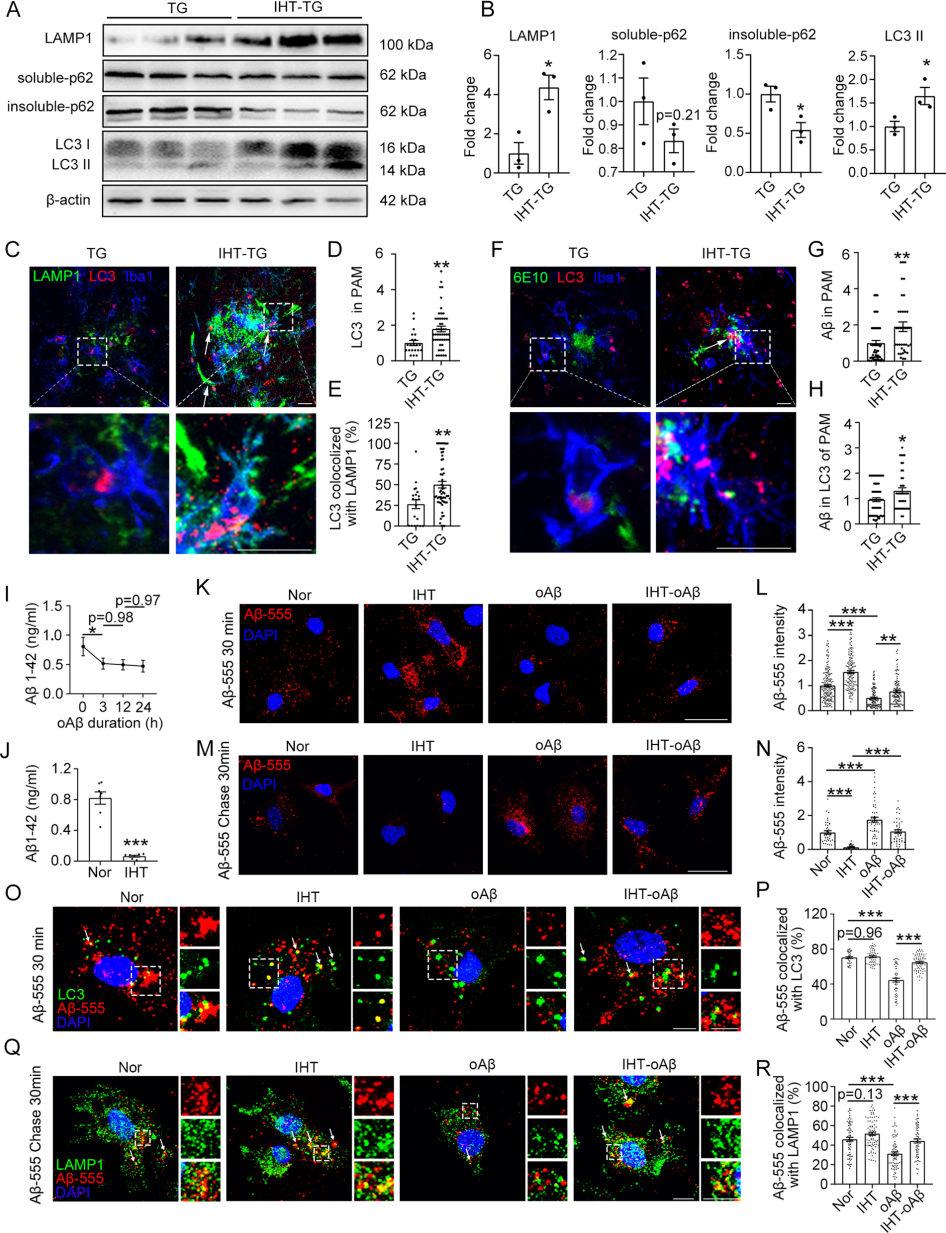
IHT enhances autophagy and Aβ degradation by autophagosomes in PAM in vivo and in vitro. **A,** **B** LAMP1, p62, and LC3 in the hippocampus were detected via western blot. n = 3 (Student's *t* test). **C**–**E** Mice brain sections were co-stained with anti-LAMP1, anti-Iba1, and anti-LC3 antibodies. Scale bar = 10 μm. LC3 accumulation, and colocalization ratio of LC3 with LAMP were quantified in Iba1^+^ cells. n > 20 (Student's *t* test). **F**–**H** Brain sections were co-stained with anti-Iba1, anti-6E10, and anti-LC3 antibodies. Scale bar = 10 μm. The amount of internalized Aβ and Aβ colocalized to LC3 puncta was quantified in Iba1^+^ cells. n > 40 (Student's *t* test). **I** Primary microglia were treated with 1 μM oAβ for the indicated durations. Aβ concentration in the medium was measured using ELISA. n = 4 (one-way ANOVA). **J** Primary microglia were treated with 1 μM oAβ for 24 h, followed by a single day of IHT (10 cycles of 21% O_2_ and 8% O_2_). Aβ concentration in the medium was determined using ELISA. n = 7 (Student's *t* test). **K**–**N** After oAβ and IHT co-treatments, primary microglia were incubated with 1 μM of Aβ-555 for 30 min (**K** and **L**), or further washed with fresh medium (Chase) for 30 min (**M** and **N**). Scale bar = 20 μm. The intracellular Aβ-555 was quantified using Image J. n > 40 (two-way ANOVA). **O**–**R** Microglia were probed with anti-LC3 or anti LAMP 1 antibodies. Scale bar = 5 μm. The ratio of Aβ-555 colocalization with LC3 or LAMP1 in a single cell was determined via Manders' colocalization coefficients. n > 50 (two-way ANOVA). *P < 0.05, **P < 0.01, and ***P < 0.00. *IHT* intermittent hypoxia therapy, *TG* APP/PS1 transgenic mice

We hypothesized that the effect of IHT on AD pathology is mediated by the upregulation of the ALP. After IHT, lysosomal-associated membrane protein 1 (LAMP1) and LC3I/II levels in the hippocampus of APP/PS1 mice were significantly upregulated and that of the insoluble content of the autophagy substrate SQSTM1/p62 was decreased, suggesting that IHT upregulates the ALP (Fig. 3A, B). Moreover, considering that Aβ clearance in the brain depends primarily on microglial autophagy [6], we further examined the effects of IHT on autophagosome formation in PAM. As shown in Fig. 3C, andD, the punctate intensities of LC3 (a well-known autophagosome marker) increased significantly in PAM after IHT. The colocalization of LC3 with LAMP1 was also increased in PAM, suggesting enhanced autolysosome formation (Fig. 3C, E). Consistent with the autophagy-dependent degradation of Aβ in PAM, elevated Aβ levels and increased colocalization of Aβ with LC3 were observed in PAM after IHT (Fig. 3F–H). These findings suggest that IHT enhances Aβ clearance by stimulating the ALP in PAM.

To further validate that IHT modulates its effects via microglia, an in vitro PAM model was established to enable the direct exposure of microglia to prolonged oAβ activation. As shown in Fig. 3I, microglia quickly degraded oAβ in the medium within 3 h of oAβ exposure, but the subsequent clearance activity was significantly diminished, suggesting that prolonged oAβ stimulation might impair microglial autophagic degradation of oAβ. Furthermore, a single day of IHT treatment (10 cycles of 8% O_2_ for 8 min and 21% O_2_ for 8 min) dramatically enhanced the clearance of the remaining oAβ by the microglia (Fig. 3J). In addition, we observed that the internalization of Aβ-555 was significantly improved in the IHT-treated PAM (Fig. 3K, L), wherein IHT was demonstrated to accelerate Aβ clearance by PAM after 30 min of intracellular degradation, i.e., chase period (Fig. 3M, N). This finding indicates that IHT enhances cellular Aβ clearance by promoting endocytosis and intracellular Aβ degradation by PAM. Furthermore, IHT significantly increased the colocalization of Aβ-555 with LC3 (Fig. 3O, P) and LAMP1 (Fig. 3Q, R), suggesting that IHT can stimulate autophagic degradation of oAβ by directly acting on microglia.

### IHT upregulates TFEB-mediated autophagy through AKT–MAPK–mTOR signaling

**Fig. 4:**
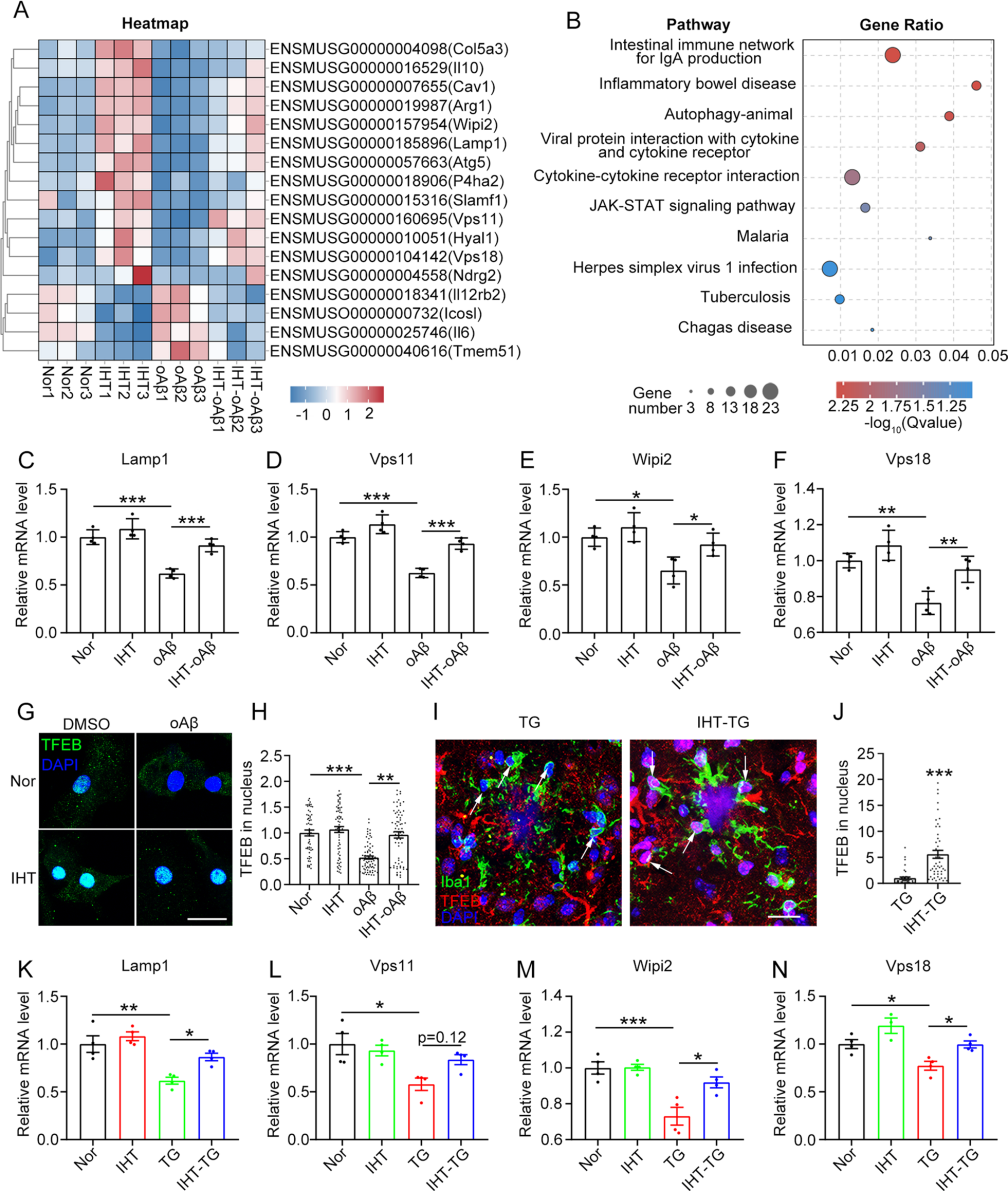
IHT upregulates the TFEB-related pathway in PAM. **A**–**F** Total RNA of in vitro PAM was isolated for RNA-seq. Heatmap **(A)** and KEGG pathway **(B)** analyses were used to demonstrate the differentially expressed genes and enriched pathways, respectively. **C**–**F** Autophagy-related differential genes, including *Lamp1*, *Vps11*, *Wipi2*, and *Vps18*, were detected using qRT-PCR. n = 3 (two-way ANOVA). **G**, **H** In vitro PAM was stained with an anti-TFEB antibody after a single day of IHT. Scale bar = 20 μm. TFEB signals in the microglial nucleus were quantified. n > 50 (two-way ANOVA). **I**, **J** Brain sections of IHT-treated mice were co-stained with anti-Iba1 and anti-TFEB antibodies. Arrows indicate the PAM nucleus. Scale bar = 20 μm. TFEB signals in the PAM nucleus were measured. n > 50 (Student's *t* test). **K**–**N** Autophagy-related genes, such as *Lamp1*, *Vps11*, *Wipi2,* and *Vps18*, in the brains of IHT-treated mice were detected via qRT-PCR. n = 3 (two-way ANOVA). *P < 0.05, **P < 0.01, and ***P < 0.001. TG, APP/PS1 transgenic mice

To explore the mechanism by which IHT upregulates oAβ degradation in PAM, we screened the PAM cells via RNA sequencing (RNA-seq). As depicted in the heatmap analysis, IHT treatment of PAM in vitro significantly increased the expression of ALP-related proteins (Fig. 4A). In addition, the levels of pro-inflammatory factors were decreased and those of anti-inflammatory factors were increased in IHT-treated PAM in vitro (Fig. 4A). Similarly, the KEGG pathway analysis revealed that the autophagy and inflammation-related pathways were among the top upregulated pathways (Fig. 4B). In particular, the mRNA levels of autophagy/lysosome-related genes, such as *Lamp1*, *Vps11*, *Wipi2*, and *Vps18*, were upregulated in the IHT-treated PAM in vitro (Fig. 4C–F).

Thus, we hypothesized that the IHT-enhanced autophagy was related to TFEB activation. In support of this hypothesis, our in vitro and in vivo results showed that the nuclear expression of TFEB in PAM was significantly increased after IHT (Fig. 4G–J). Moreover, IHT upregulated the transcription activity of TFEB (Additional file 1: Figs. S3 and S6). In consistent, IHT significantly upregulated the expression of ALP-related genes in mice brains, including *Lamp1*, *Vps11*, *Wipi2*, and *Vps18*, which were also target genes of TFEB (Fig. 4K–N). Next, to address the mechanism of TFEB activation by IHT in PAM, we investigated the activities of AKT, MAPK, and mTOR signaling, which serve as the negative regulators of TFEB activation. Our results demonstrated that IHT inhibited the phosphorylation levels of AKT, MAPK, mTOR, and RPS6 in vivo (Additional file 1: Fig. S4A, B) and in vitro (Additional file 1: Fig. S4C, D), indicating that IHT enhanced TFEB activation in PAM by inhibiting AKT–MAPK–mTOR signaling. These results suggest that IHT-enhanced autophagic degradation of Aβ by PAM is associated with the upregulation of ALP-related proteins, which in turn is mediated by TFEB activation.

### TFEB activation ameliorates Aβ pathology and enhances Aβ autophagic degradation by PAM in vivo and in vitro

**Fig. 5:**
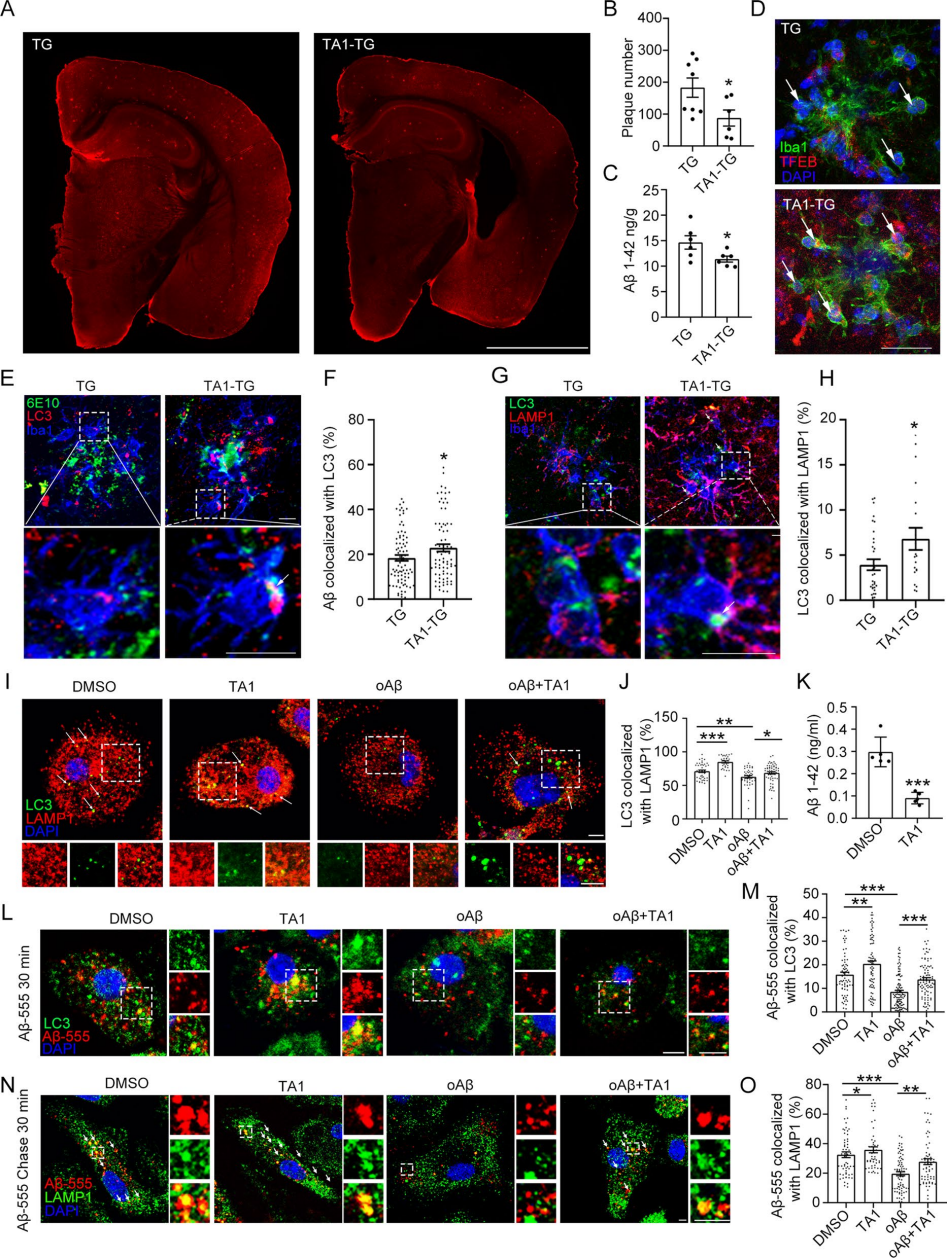
TA1 enhances the autophagic degradation of Aβ and reduces Aβ pathology in APP/PS1 mice. 6-month-old APP/PS1 mice were administered TA1 orally at 10 mg/kg for 3 months. **A**, **B** Mice brain sections were probed with anti-Aβ1–16 (6E10) antibodies. Scale bar = 2 mm. The number of hippocampal and cortical plaques was quantified in twelve evenly spaced brain slices from each mouse. n = 8, 6 (Student's *t* test). **C** Aβ1–42 in brain tissues was determined using ELISA. n = 6 (Student's *t* test). **D** Brain sections were co-stained with anti-TFEB and anti-Iba1 antibodies. Arrows indicate the PAM nuclei. Scale bar = 25 μm. **E**–**H** Brain sections were co-stained with anti-LC3, anti-Iba1, and anti-Aβ1–16 (6E10) or anti-LAMP1 antibodies. Scale bar = 10 μm. The colocalization ratio of LC3 with Aβ or LAMP1 in Iba1^+^ cells were estimated via Manders' colocalization coefficients. n > 30 (Student's *t* test). **I**, **J** Primary microglia were co-treated with oAβ and TA1 for 12 h, followed with bafilomycin A1 treatment for 1 h. Then, cells were probed with anti-LC3 and anti-LAMP1 antibodies. Scale bar = 5 μm. The colocalization ratio of LC3 with LAMP1 in single cell was quantified using Manders' colocalization coefficients. n > 40 (two-way ANOVA). **K** Aβ1–42 in the medium were then detected using ELISA. n = 5 (Student’s *t* test). **L**–**O** After co-treated with oAβ and TA1, cells were incubated with Aβ-555 for 30 min, or co-treated with bafilomycin A1 for 30 min and chased. Then, cells were stained with anti-LC3 or anti-LAMP1 antibodies. Scale bar = 5 μm. The colocalization ratio of Aβ-555 with LC3 or LAMP1 was measured via Manders’ colocalization coefficients. n > 50 (two-way ANOVA). *P < 0.05, **P < 0.01, and ***P < 0.001. TG, APP/PS1 transgenic mice

TA1, also named as Curcumin analog C1, was reported as a TFEB-specific activator by binding to the N-terminus of TFEB, promoting TFEB nuclear translocation and activating TFEB-mediated autophagy [41], which has no effect on the pathways regulating autophagy, including mTOR, MAPK1/ERK2 and MAPK3/ERK1. TA1 has been reported to reduce Aβ pathology and improve cognitive function by effectively activating TFEB in the brain of 3xTg AD model mice [31]. In our study, TA1induced TFEB nuclear translocation in primary microglial cells (Additional file 1: Fig. S5). Thus, we further examined whether TA1 can alleviate AD pathology by activating the TFEB pathway in the PAM of APP/PS1 mice. Our findings demonstrated that TA1 reduced Aβ plaques in the mouse brain (Fig. 5A, B) and Aβ load in the cortex (Fig. 5C). In addition, TA1 increased the transcription activity of TFEB (Additional file 1: Fig. S6) and nuclear translocation of TFEB in PAM (Fig. 5D), which was in line with TA1's established role in TFEB activation [41]. We also revealed that TA1 upregulated the colocalization of Aβ with LC3 as well as the colocalization of LC3 with LAMP1 in PAM (Fig. 5E–H), consistent with the observation of TFEB-enhanced autophagic degradation of Aβ.

Furthermore, we examined whether TA1 directly affects microglia. For this purpose, we co-treated microglia with TA1 and oAβ. Our results showed that TA1 upregulated the nuclear levels of TFEB (Additional file 1: Fig. S7A, B). In consistent, TA1 upregulated the transcription activity of TFEB (Additional file 1: Fig. S3) and its target genes *Lamp1*, *Vps11*, *Wipi2,* and *Vps18* in PAM in vitro (Additional file 1: Fig. S7C–F). TA1 was also demonstrated to upregulate the colocalization of LC3 with LAMP1 (Fig. 5I, J) and enhance Aβ clearance by microglia (Fig. 5K), indicating that TFEB activation contributed to oAβ degradation. Furthermore, TA1 increased the colocalization ratio of Aβ-555 with both LC3 and LAMP1 in PAM in vitro (Fig. 5L–O). These findings suggested that TFEB activation by TA1 enhanced Aβ degradation via the upregulation of the ALP in PAM in vitro, supporting the in vivo results*.*

### IHT enhances TFEB-mediated ALP-dependent Aβ degradation in microglia

**Fig. 6:**
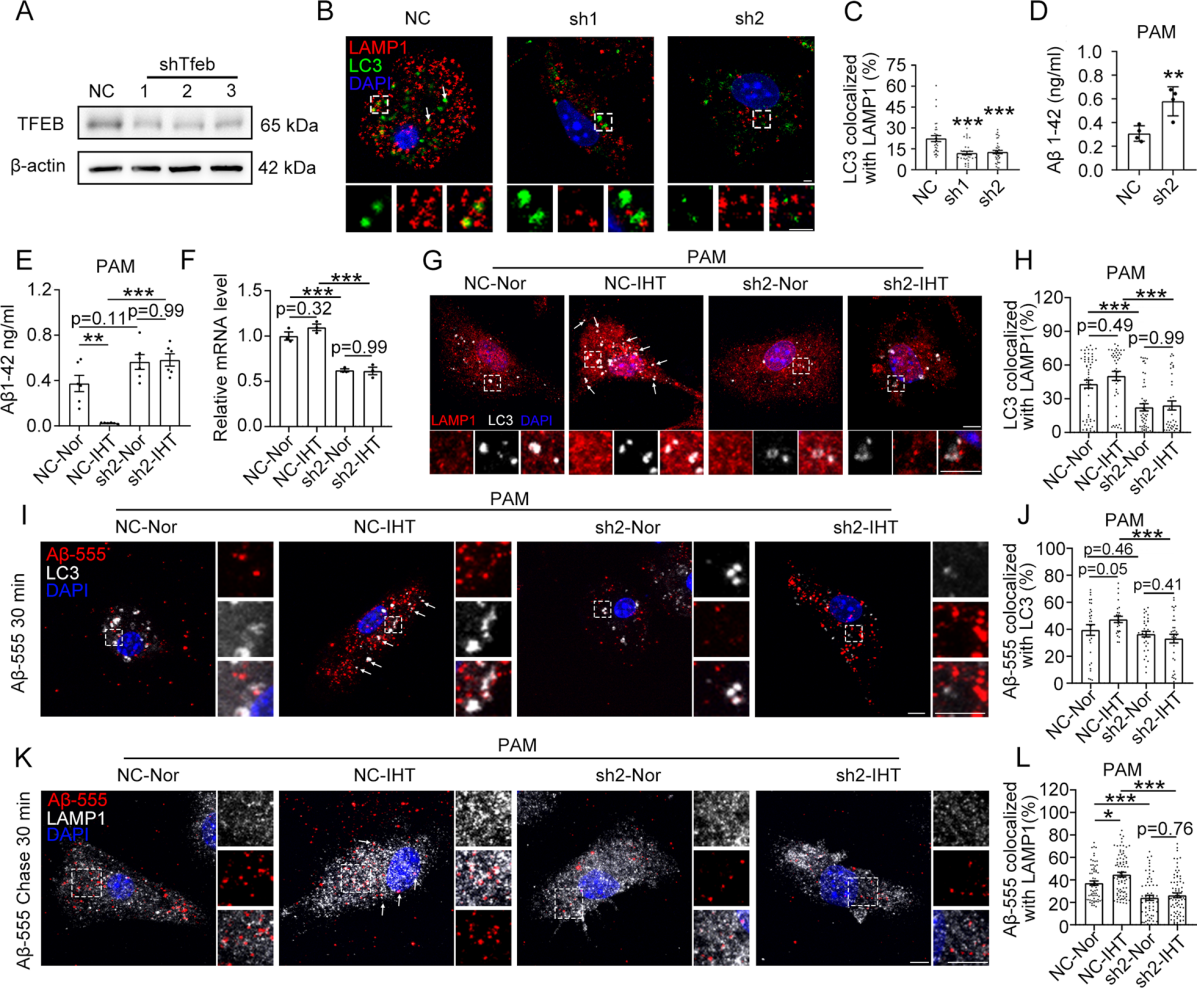
IHT enhances Aβ degradation by microglia via TFEB-mediated autophagy. **A** Primary microglia were infected with three shTfeb lentivirus (#1, #2, and #3). Interference efficiency were determined by western blot. **B**, **C** Cells were co-stained with anti-LC3 and anti-LAMP1 antibodies. Scale bar = 5 μm. The colocalization ratio of LC3 and LAMP1 in single cell was quantified using Manders' colocalization coefficients. n > 30 (Student's *t* test). **D** sh-Tfeb #2 lentivirus (sh2)-infected microglia were treated with 1 μM of oAβ for 12 h. Aβ1–42 in the medium were detected with ELISA. n = 4. (Student's *t* test). **E**–**L** sh-Tfeb #2 lentivirus (sh2)-infected microglia were co-treated with oAβ and a single-day IHT. **E** Aβ1–42 in the medium was measured via ELISA. n = 6. **F** TFEB expression was determined using qRT-PCR. n = 3. **G**, **H** Cells were treated with bafilomycin A1 for 1 h, followed by staining with anti-LC3 and anti-LAMP1 antibodies. Scale bar = 5 μm. The colocalization ratio of LC3 with LAMP1 was evaluated using Manders' colocalization coefficients. n > 40. **I**–**L** Cells were treated with Aβ-555 for 30 min, or co-treated with Aβ-555 and bafilomycin A1 for 30 min and chased. Then, cells were stained with anti-LC3 or anti-LAMP1 antibodies. Scale bar = 5 μm. The colocalization ratio of Aβ-555 with LC3 or LAMP 1 in a single cell was determined via Manders' colocalization coefficient. n > 30. *P < 0.05, **P < 0.01, and ***P < 0.001 using two-way ANOVA

To determine the role of TFEB in the autophagic degradation of Aβ by microglia, we inhibited TFEB expression by infecting primary microglial cells with lentivirus expressing sh*Tfeb* (#1, #2, and #3) (Fig. 6A). Our results showed that the colocalization of LC3 with LAMP1 was significantly decreased after TFEB silencing (Fig. 6B, C). Furthermore, oAβ degradation by microglia was impaired after TFEB knockdown (Fig. 6D), indicating that TFEB is important for Aβ degradation by microglia.

To further investigate the involvement of TFEB in IHT-enhanced Aβ degradation by PAM, we silenced TFEB in IHT-treated PAM in vitro. We found that TFEB silencing (Fig. 6F) inhibited the IHT-associated improvement of oAβ clearance by microglia (Fig. 6E). Similar to the findings in the primary microglial cells, the absence of TFEB in IHT-treated PAM in vitro prevented the enhanced colocalization of LC3 with LAMP1 (Fig. 6G, H), suggesting that IHT upregulated the ALP via TFEB. Furthermore, TFEB interference restricted IHT-stimulated augmentation of Aβ-555 colocalization with both LC3 and LAMP1 in PAM in vitro (Fig. 6I–L). Thus, the effect of IHT on the ALP-dependent degradation of Aβ in PAM is mediated by TFEB signaling.

## Discussion

Intermittent hypoxia can have contrasting effects on the central nervous system based on the frequency, degree, and duration of hypoxia exposure [42]. Intermittent hypoxic conditions of high intensity, high density, and long periods are known to damage the central nervous system and are commonly used to simulate sleep apnea [43]. In contrast, intermittent hypoxic stimulation of appropriate intensity exerts a protective effect. Ryou et al. demonstrated that 3 weeks of IHT effectively stabilized neurological function and upregulated neuroprotective trophic factors and erythropoietin in 3xTg-AD model mice, significantly protecting the AD model mice from cognitive impairment [33]. Yue et al. found that IHT with alternately 10 min of 14.3% O_2_ and 10 min of 21% O_2_, 4 h daily for 14 days, significantly reduced the level of Aβ plaque and nerve fiber accumulation in the brain of 6-month-old APP/PS1 mice and improve their learning and memory ability [44]. Meng et al. found that IHT with 8 min of 5% O_2_ and 8 min 21% O_2_ alternately for 4 h every day for 15 days, significantly improved the cognitive impairment of 9-month-old APP/PS1 mice [45]. It was reported that IHT lasting for 28 days or more also showed protective effect. After IHT at an altitude of 7000 m (about 8% O_2_) for 8 h per day for 35 days, the infarcted area of the brain reduced and the arrhythmia caused by coronary artery occlusion was improved [46]. Giving 14% O_2_ for 12 h a day for 28 days effectively improved the glucose tolerance of diabetic rats [47]. In addition to the hypoxic conditions used in this study, we also treated mice with different levels of intermittent hypoxia, including 5% O_2_ for 30 s, 21% O_2_ for 30 s with 8 h treatment daily for 28 days, and 12% O_2_ for 8 min, 21% O_2_ for 8 min with 10 cycles treatment daily for 28 days. We found that the above conditions had no obvious effect on the learning and memory ability of APP/PS1 mice (data not shown), suggesting that the protective effect of IHT on AD mice depends on different hypoxia conditions. The clinical evidence shows that in the past 50 years, IHT with the concentration of 10–15% and the duration of 3 weeks with head-mounted or walk-in equipment produce significant beneficial effects in the treatment of cardiovascular diseases, such as hypertension, coronary heart disease and heart failure [48], proving that IHT has a good prospect in clinic.

In the present study, we found no significant improvement in anxiety by IHT. The relationship between AD and anxiety remains unclear. A study by Hanseeuw et al. showed that, in the early stages of AD, patients did not show anxiety, and in the late stages of AD, anxiety gradually appeared [49]. Zhang et al. demonstrated that Citalopram improved spatial memory and synaptic plasticity in the hippocampus of AD mice, but did not positively affect anxiety-like behavior [50]. Similarly, Xu et al. found that ketogenic diet therapy effectively improved learning cognitive ability and significantly reduced the number of Aβ plaques and neuroinflammation in the brain, but did not have a significant improvement in anxiety-like behavior [51]. We hypothesized that the improvements in cognitive functioning would precede the onset of improvements in anxiety.

Our study investigated the modulatory effect of IHT on TFEB-associated autophagy in the PAM of AD mice. Previous literature suggests that the beneficial effects of IHT pretreatment may be related to the optimization of mitochondrial metabolism and the prevention of ROS overproduction [23]. Clinical and basic studies have also revealed that autophagy is significantly reduced in the brains of patients with AD and that Aβ degradation in the brain mainly depends on autophagic degradation by microglia [10]. Furthermore, impaired phagocytosis and autophagy in PAM were identified as the major factors involved in plaque deposition, neuronal apoptosis, and neuroinflammation in the advanced AD brain [12, 13]. Nevertheless, the reactivation of autophagic activities has shown positive outcomes in AD pathology [52, 53]. In the present study, we determined that IHT effectively stimulated autophagy in PAM and alleviated AD pathology, enhancing the microglial function of clearing Aβ and reducing Aβ plaque deposition. Therefore, IHT has promising potential as an adjuvant treatment for AD.

TFEB abnormalities are closely associated with many diseases [15]. Moreover, TFEB is known to promote the transcriptional upregulation of lysosome/autophagy genes by directly binding to the Coordinated Lysosomal Expression and Regulation (CLEAR) elements in their promoters [54, 55]. The CLEAR network genes are involved in autophagosome and lysosomal biogenesis, lysosomal exocytosis, endocytosis, and membrane repair [54, 55]. Furthermore, the activation of neurological TFEB has been reported to regulate microglia-mediated degradation of Aβ plaques via the ALP [19, 21]. In our investigation, we found that the attenuation of AD pathology by IHT was associated with upregulated TFEB function and suppressed mTORC1 signaling. In addition, IHT upregulated TFEB nuclear translocation in PAM and improved the autophagic clearance of Aβ. Consistent with our findings, TFEB activation has been previously shown to increase Aβ clearance and lysosomal biogenesis in neurons and astrocytes in APP/PS1 mice [56]. In addition to mTORC1-related TFEB activation, SIRT1-induced TFEB deacetylation also enhances microglial phagocytosis of Aβ and lysosomal biogenesis [57]. Thus, increased TFEB activation in microglia leads to upregulated autophagic/lysosomal clearance of Aβ, thereby reducing the Aβ load in the brain.

An interesting thing was that the nuclear translocation of TFEB caused by TA1 was not as significant as that caused by IHT in PAM, but it still caused a significant reduction of Aβ plaque. Considering that TFEB is widely expressed in the central nervous system, especially in neurons, upregulation of TFEB also enhances autophagy of APP and reduce the release of Aβ [58, 59]. It might contribute to the decrease of Aβ plaque. TA1 is used in vivo at a dose of 10 mg/kg. Song et al. proved that 10 mg/kg TA1 could be used for the treatment of AD mice [31]. He et al. also used the same dosage in studying the damage of sensory hair cells in mice [60]. Interestingly, Fig. S5 also shows that TA1 has the effect of decreasing nuclear translocation at high concentrations, suggesting that a lower concentration of TA1 may induce a higher TFEB nuclear translocation of PAM.

During late-stage AD, autophagy is typically downregulated in PAM [13, 53, 61]. Prolonged oAβ exposure has the opposite effect by impairing autophagy [62, 63]. Thus, we hypothesized that prolonged oAβ stimulation causes the pro-inflammatory polarization of microglia via autophagic overload, thereby impairing microglia autophagy. After treating microglia with different durations of oAβ stimulation, we observed that the microglial ability to degrade Aβ was significantly impaired after 12 h. Increasing research indicates that the inhibition of TFEB kinases [64–66], activation of TFEB acetylation [21], and mTOR-dependent or -independent pharmacological activation of TFEB [52, 67, 68] have therapeutic potential in AD animal models. Thus, targeting TFEB may be a promising strategy for AD treatment. In addition, many studies have demonstrated the beneficial role of IHT in treating multi-organ diseases, suggesting that IHT may serve as an adjunctive treatment for AD. However, the appropriate IHT parameters should be investigated further to achieve optimal benefits and reduce potential risks. Whether IHT has harmful or beneficial effects on other functional cells of the brain is an important reference for examining whether IHT would be available as a treatment for potential AD disorders. Therefore, the study of IHT on a variety of other cells of the nervous system deserves to be initiated.

## Conclusion

The results of the present study revealed that IHT alleviates AD pathology by activating TFEB in PAM, leading to the transcriptional upregulation of the ALP that in turn improves microglia-mediated Aβ clearance in the brain of AD mice. This underlying mechanism highlights the potential of TFEB as a therapeutic target for AD treatment. Furthermore, compared with small-molecule TFEB activators, IHT may be a promising alternative therapeutic approach for TFEB activation that also avoids the undesired side effects of chemical drugs.

### Supplementary Information

**Additional file 1: Figure S1.** Anxiety of APP/PS1 mice was not significantly improved after IHT. 8-Month-old APP/PS1 mice were treated with IHT for 28 days followed with EPM test (A to C) or OFT (D to F). (A) The trajectory of mice in EPM test for 5 min. (B) The ratio of open arm distance was calculated by the open arm distance/total distance × 100%. (C) Time spent in open arms during EPM test. (D) The trajectory of mice in OFT for 5 min. (E) Entries to center area (small square area) in 5 min test duration. (F) Time spent in center area during OFT. n = 6, * *P* < 0.05, ** *P* < 0.01 and *** *P* < 0.001 by student *t* test. TG, APP/PS1 transgenic mice. **Figure S2.** IHT reduced synaptic loss in APP/PS1 mice. 8-Month-old APP/PS1 mice were treated with IHT for 28 days. (A) Brain sections of IHT mice were stained with anti–anti-synaptophysin (SYP) antibody (Abcam, ab8049) to label synaptosome. Scale bar = 100 μm. The synaptosome content were obtained by calculating the relative area of SYP signals in cortex (B) hippocampal CA1 region (C). n = 6, * *P* < 0.05, ** *P* < 0.01 and *** *P* < 0.001 by two-way ANOVA. Ctx, cortex; Hipp, hippocampus. TG, APP/PS1 transgenic mice. **Figure S3.** IHT activates TFEB transcription activity. A Luciferase reporter system containing three CLEAR elements in the promoter region was constructed and transfected to HEK393T cells. Luciferase activity was determined after IHT or TA1 treatment. pGL-Basic was the empty plasmid. n = 3, * *P* < 0.05, ** *P* < 0.01 and *** *P* < 0.001 by student *t* test. **Figure S4.** IHT inhibits the TFEB upstream AKT–MAPK–mTOR signaling in PAM. (A and B) Proteins of mTOR, AKT, MAPK, and RPS6 and their phosphorylation levels in the hippocampus of IHT-treated APP/PS1 mice were detected by Western blot and quantified. n = 3 (student *t* test). (C and D) Proteins of mTOR, Akt, MAPK, and RPS6 and their phosphorylation levels of IHT-treated-in vitro PAM were detected by Western blot and quantified. n = 3 (two-way ANOVA). * *P* < 0.05 and *** *P* < 0.001 TG, APP/PS1 transgenic mice. **Figure S5.** Low concentration TA1 promoted nuclear translocation of TFEB. Primary microglia were treated with TA1 at indicated concentration for 12 h. TFEB was labeled by anti-TFEB antibody. Scale bar = 50 μm. The nuclear translocation of TFEB was calculated by quantifying the TFEB intensity in DAPI positive region. At least 50 cells were counted per treatment. ** *P* < 0.01 and *** *P* < 0.001 by one-way ANOVA. **Figure S6.** IHT/TA1 activates enrichment of CLEAR element by TFEB in APP/PS1 mice brain. (A) The position indicates, where the element is located from the transcription start site. (B) Anti-TFEB antibody was used for the Chromatin Immunoprecipitation assay and quantification of immunoprecipitated DNA fragments was performed by PCR. The PCR product was obtained from the chromatin without immunoprecipitation reaction as group Input and IgG was as negative control. (C) Enrichments of the Vps35 promoter by TFEB in IHT or TA1 treated brain tissue were measured by qPCR. * *P* < 0.05 and ** *P* < 0.01 by student *t* test. **Figure S7.** TA1 enhanced TFEB nuclear translocation and upregulate the mRNA levels of TFEB target genes in vitro PAM model. Primary microglia were co-treated with 1 μM oAβ and 1 μM TA1 for 12 h. (A, B) Cells were fixed and stained with anti-TFEB antibody and DAPI. Nuclear translocation of TFEB was measured by quantifying the intensity of TFEB in DAPI. Scale bar = 25 μm. n > 50. (C–F) mRNA levels of *Lamp1*, *Vps11*, *Wipi2*, and *Vps18* were determined by qRT-PCR. n = 3. * *P* < 0.05, ** *P* < 0.01 and *** *P* < 0.001 by two-way ANOVA.

## Data Availability

The authors confirm that the data supporting the findings of this study are available within the article and its Supplementary material. Raw data that support the findings of this study are available from the corresponding author, upon reasonable request.

## Abbreviations

Aβ: Beta-amyloid
AD: Alzheimer's disease
ALP: Autophagy–lysosomal pathway
Aβ-555: HiLyte™ Fluor 555 labeled oligomer Aβ1-42
CLEAR: Coordinated Lysosomal Expression and Regulation
IHT: Intermittent hypoxia therapy
LANDO: LC3-associated endocytosis
LAMP1: Lysosomal-associated membrane protein 1
MAPK: Mitogen-activated protein kinase
mTOR: Mechanistic target of rapamycin kinase
MWM: Morris water maze
oAβ: Aβ oligomer
PAM: Plaque-associated microglia
RPS6: Ribosomal protein S6
SEM: Standard error of measurement
TFEB: Transcription factor EB
TA1: TFEB activator 1
TG: APP/PS1 transgenic mouse
WT: Wide type
