# Supplementary material for: Intermittent hypoxia therapy ameliorates beta-amyloid pathology via TFEB-mediated autophagy in murine Alzheimer's disease
Source: J Neuroinflammation. 2023 Oct 20;20:240. doi: 10.1186/s12974-023-02931-6 (PMC10588168; doi:10.1186/s12974-023-02931-6)
Supplement: Supplementary file 1 — Additional file 1: Figure S1. Anxiety of APP/PS1 mice was not significantly improved after IHT. 8-Month-old APP/PS1 mice were treated with IHT for 28 days followed with EPM test (A to C) or OFT (D to F). (A) The trajectory of mice in EPM test for 5 min. (B) The ratio of open arm distance was calculated by the open arm distance/total distance × 100%. (C) Time spent in open arms during EPM test. (D) The trajectory of mice in OFT for 5 min. (E) Entries to center area (small square area) in 5 min test duration. (F) Time spent in center area during OFT. n = 6, * P < 0.05, ** P < 0.01 and *** P < 0.001 by student t test. TG, APP/PS1 transgenic mice. Figure S2. IHT reduced synaptic loss in APP/PS1 mice. 8-Month-old APP/PS1 mice were treated with IHT for 28 days. (A) Brain sections of IHT mice were stained with anti–anti-synaptophysin (SYP) antibody (Abcam, ab8049) to label synaptosome. Scale bar = 100 μm. The synaptosome content were obtained by calculating the relative area of SYP signals in cortex (B) hippocampal CA1 region (C). n = 6, * P < 0.05, ** P < 0.01 and *** P < 0.001 by two-way ANOVA. Ctx, cortex; Hipp, hippocampus. TG, APP/PS1 transgenic mice. Figure S3. IHT activates TFEB transcription activity. A Luciferase reporter system containing three CLEAR elements in the promoter region was constructed and transfected to HEK393T cells. Luciferase activity was determined after IHT or TA1 treatment. pGL-Basic was the empty plasmid. n = 3, * P < 0.05, ** P < 0.01 and *** P < 0.001 by student t test. Figure S4. IHT inhibits the TFEB upstream AKT–MAPK–mTOR signaling in PAM. (A and B) Proteins of mTOR, AKT, MAPK, and RPS6 and their phosphorylation levels in the hippocampus of IHT-treated APP/PS1 mice were detected by Western blot and quantified. n = 3 (student t test). (C and D) Proteins of mTOR, Akt, MAPK, and RPS6 and their phosphorylation levels of IHT-treated-in vitro PAM were detected by Western blot and quantified. n = 3 (two-way ANOVA). * P < 0.05 and *** P < 0.001 T [file 12974_2023_2931_MOESM1_ESM.docx]

**Additional Figures**


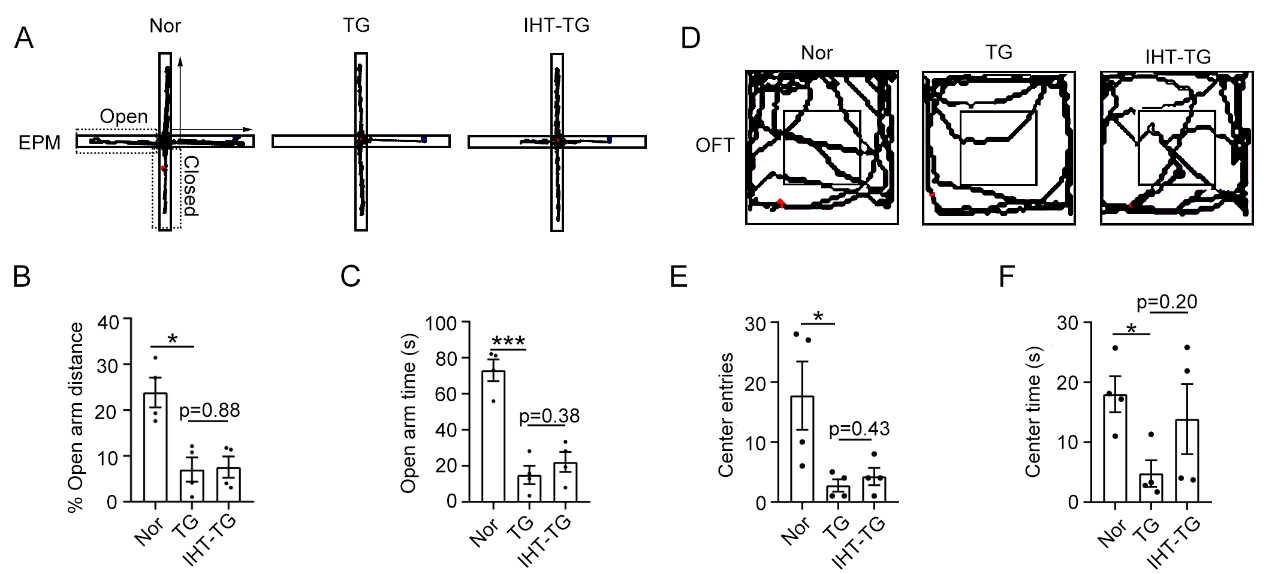


**Figure S1 The anxiety of APP/PS1 mice was not significantly improved after IHT.** 8-month-old APP/PS1 mice were treated with IHT for 28 days followed with EPM test **(A to C)** or OFT **(D to F)**. **(A)** The trajectory of mice in EPM test for 5 min. **(B)** The ratio of open arm distance was calculated by the open arm distance/total distance × 100%. **(C)** Time spent in open arms during EPM test. **(D)** The trajectory of mice in OFT for 5 min. **(E)** Entries to center area (small square area) in 5 mins-test duration. **(F)** Time spent in center area during OFT. n = 6, * *P* < 0.05, ** *P* < 0.01 and *** *P* < 0.001 by student t-test. TG, APP/PS1 transgenic mice.


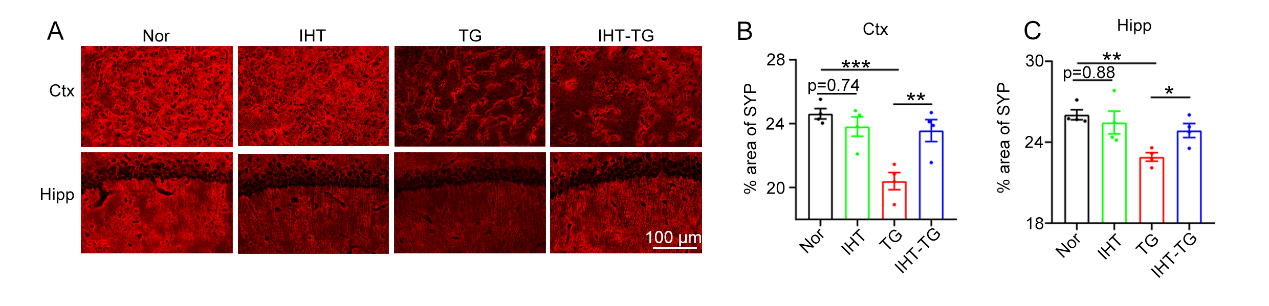


**Figure S2 IHT reduced synaptic loss in APP/PS1 mice.** 8-month-old APP/PS1 mice were treated with IHT for 28 days. **(A)** Brain sections of IHT mice were stained with anti-anti-synaptophysin (SYP) antibody (Abcam, ab8049) to label synaptosome. Scale bar = 100 μm. The synaptosome content were obtained by calculating the relative area of SYP signals in cortex **(B)** hippocampal CA1 region **(C)**. n = 6, * *P* < 0.05, ** *P* < 0.01 and *** *P* < 0.001 by two-way ANOVA. Ctx, cortex; Hipp, hippocampus. TG, APP/PS1 transgenic mice.


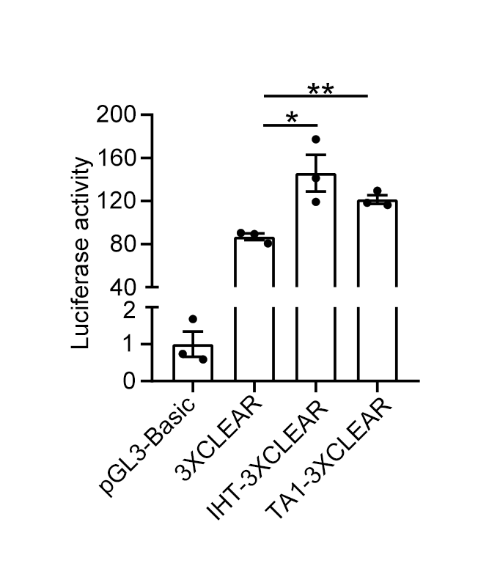


**Figure S3 IHT activates TFEB transcription activity.** A Luciferase reporter system containing three CLEAR elements in the promoter region was constructed and transfected to HEK393T cells. Luciferase activity was determined after IHT or TA1 treatment. pGL-Basic was the empty plasmid. n = 3, * *P* < 0.05, ** *P* < 0.01 and *** *P* < 0.001 by student t-test.


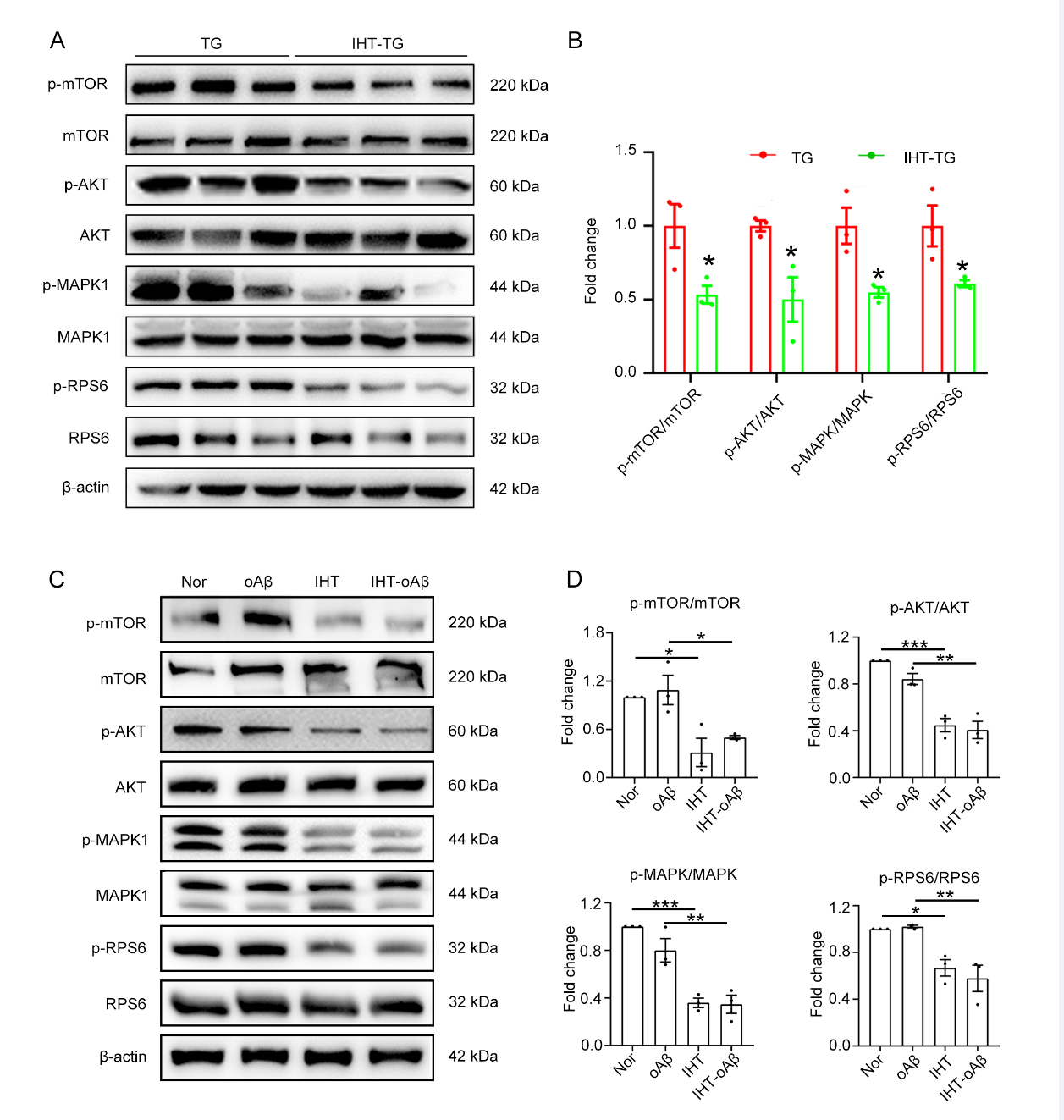


**Figure S4** **IHT inhibits the TFEB upstream AKT -MAPK-mTOR signaling in PAM. (A and B)** Proteins of mTOR, AKT, MAPK, and RPS6 and their phosphorylation levels in the hippocampus of IHT-treated APP/PS1 mice were detected by Western blot and quantified. n = 3 (student t-test). **(C and D)** Proteins of mTOR, Akt, MAPK, and RPS6 and their phosphorylation levels of IHT-treated-in vitro PAM were detected by Western blot and quantified. n = 3 (two-way ANOVA). * *P* < 0.05 and *** *P* < 0.001 TG, APP/PS1 transgenic mice.


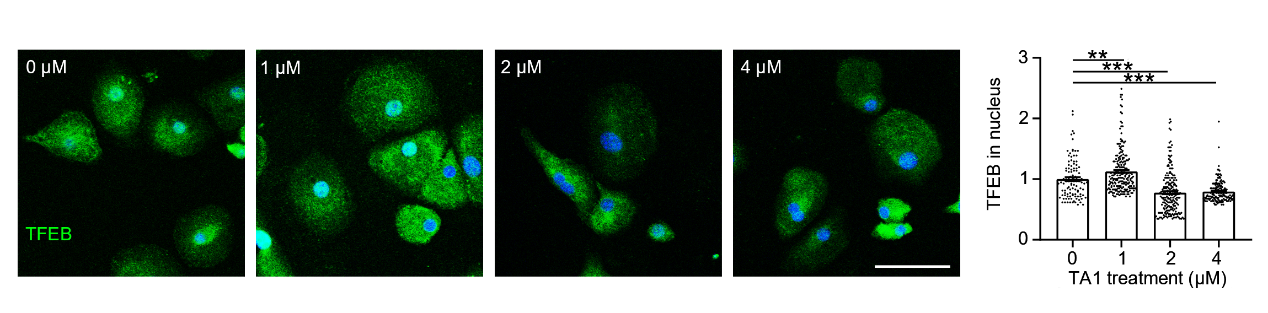


**Figure S5 Low concentration TA1 promoted nuclear translocation of TFEB.** Primary microglia were treated with TA1 at indicated concentration for 12 h. TFEB was labeled by anti-TFEB antibody. Scale bar = 50 μm. The nuclear translocation of TFEB was calculated by quantifying the TFEB intensity in DAPI positive region. At least 50 cells were counted per treatment. ** *P* < 0.01 and *** *P* < 0.001 by one-way ANOVA.


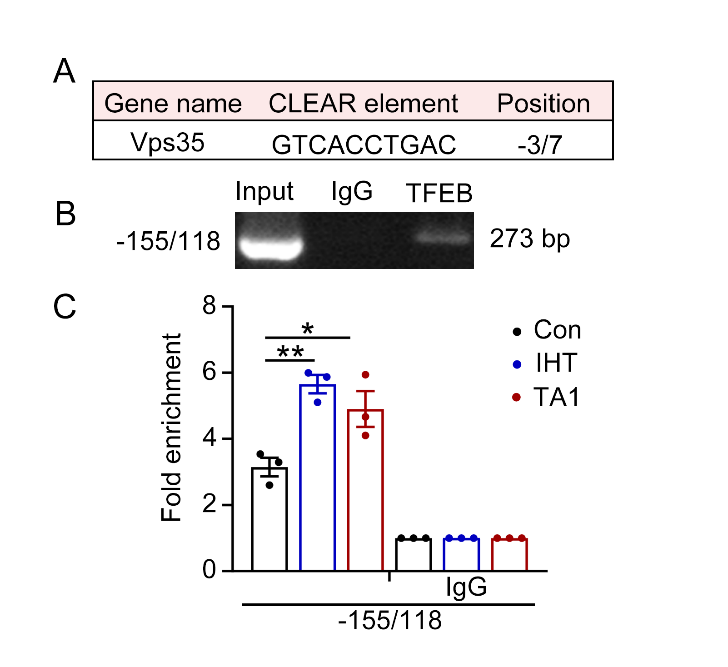


**Figure S6 IHT /TA1 activates enrichment of CLEAR element by TFEB in APP/PS1 mice brain.** **(A)** The position indicates where the element is located from the transcription start site. **(B)** Anti-TFEB antibody was used for the Chromatin Immunoprecipitation assay and quantification of immunoprecipitated DNA fragments was performed by PCR. The PCR product was obtained from the chromatin without immunoprecipitation reaction as group Input and IgG was as negative control. **(C)** Enrichments of the Vps35 promoter by TFEB in IHT or TA1 treated brain tissue were measured by qPCR. * *P* < 0.05 and ** *P* < 0.01 by student t-test.


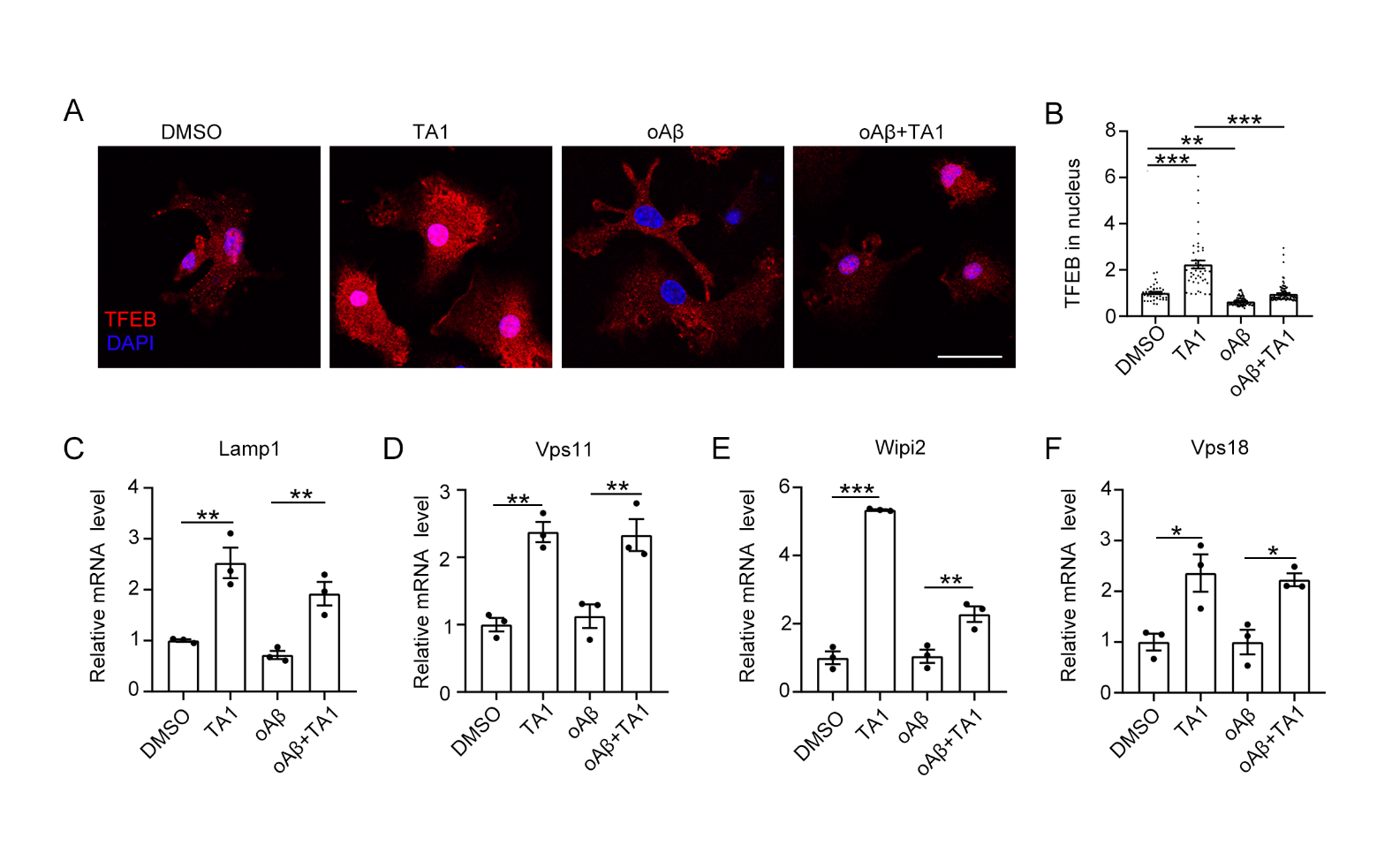


**Figure S7 TA1 enhanced TFEB nuclear translocation and upregulate the mRNA levels of TFEB target genes in vitro PAM model.** Primary microglia were co-treated with 1 μM oAβ and 1 μM TA1 for 12 h. **(A-B)** Cells were fixed and stained with anti-TFEB antibody and DAPI. Nuclear translocation of TFEB was measured by quantifying the intensity of TFEB in DAPI. Scale bar = 25 μm. n > 50. **(C-F)** The mRNA levels of *Lamp1*, *Vps11*, *Wipi2*, and *Vps18* were determined by qRT-PCR. n = 3. * *P* < 0.05, ** *P* < 0.01 and *** *P* < 0.001 by two-way ANOVA.
